# Anesthetic Management of a Patient With Common Carotid Artery Stenosis Undergoing Laryngeal Carcinoma Surgery: A Case Report

**DOI:** 10.7759/cureus.23356

**Published:** 2022-03-21

**Authors:** Purva C Shah, Chetan K Shah, Himanshu Jindal

**Affiliations:** 1 Department of Internal Medicine, Baroda Medical College, MS University, Vadodara, IND; 2 Department of Anesthesiology, HCG Cancer Hospital, Vadodara, IND; 3 Department of Internal Medicine, Ganesh Shankar Vidyarthi Memorial Medical College, Kanpur, IND

**Keywords:** carotid artery block, laryngeal carcinoma, general anesthesia, laryngeal neoplasm, carotid artery stenosis

## Abstract

Laryngectomy is a common surgery for an oncosurgeon, but underlying carotid compromise is a serious concern for anesthesiologists, making this routine procedure a high-risk one. The utmost vigilance of the anesthesiologist is demanded by the surgery to prevent morbidities such as hemiplegia, hemiparesis, or speech abnormalities that may occur due to perfusion insufficiency secondary to the mechanical blockage of the carotid arteries. Hence, an undiagnosed case of carotid artery block may result in disastrous consequences for the patient, surgeon, and anesthesiologist. Hence, it is imperative to perform all the pre-operative investigations with due diligence. We present the case of a 74-year-old male who was admitted to our set-up for laryngeal carcinoma surgery. The patient had received chemoradiotherapy (CRT) six months earlier. He complained of hoarseness in his voice and a painless neck mass. He was a known case of hypertension for 14 years, controlled by oral medication, and had a history of stroke five years ago, when he was also diagnosed with a completely blocked right common carotid artery (CCA) and a partially blocked left common carotid artery.

## Introduction

Carotid artery stenosis is found in 10-20% of all patients suffering from an ischemic stroke [1]. Studies show that, out of all the co-morbidities, arterial stenosis is the one that is most directly related to a higher risk of stroke. Hence, the primary objective of all diagnostic tests and strategies must be to ascertain the degree of stenosis [2]. The North American symptomatic carotid endarterectomy trial (NASCET) states that in patients who are treated conservatively for carotid artery stenosis, the ipsilateral recurrence of stroke is 4.4% and 13% for stenosis occluding 50-69% and 70% of the arterial lumen, respectively. In asymptomatic patients, the risk of getting a stroke is 1-2% for 60% stenosis. However, the risk increases with age, occlusion of the contralateral artery, poor collateral circulation, evidence of silent embolization, coronary artery disease, longstanding inflammatory state, or peripheral artery disease [3].

Administering general anesthesia in a patient with carotid artery stenosis, on one hand, allows us to control the ventilation and airway of the patient, while on the other hand, anesthetic agents reduce the sympathetic and baroreceptor activity, resulting in an alleviation in cardiac output and blood pressure. In addition to this, the anesthetic depth may mask intraoperative neurological complications [4,5]. Some studies proposed the ability of hypercapnia to cause cerebral vasodilation and increase cerebral blood flow; however, it may lead to cerebral vascular steal phenomenon causing adverse effects [6-8]. Propofol is the most widely used induction agent; however, it causes hypotension by reducing sympathetic outflow and producing a negative inotropic effect, among other mechanisms. Thiopental causes a milder reduction in cardiac output and blood pressure, due to which it is preferred over propofol in cases where severe hypotension is to be prevented [9,10]. In addition to this, thiopental shows neuroprotective effects against hypoxia, inflammation, degeneration, and energy failure. Ketamine and nitrous oxide (N_2_O) increase brain metabolism and, consequently, oxygen consumption, while the former also interacts with neurological monitoring. Hence, both of these anesthetic agents must be avoided in patients with already compromised cerebral blood flow [11]. Studies have revealed that a combination of sevoflurane, propofol, and N_2_O perturbs cerebral homeostasis and vascular reactivity [12].

## Case presentation

A 74-year-old married male with a body mass index of 20.11 kg/m^2^, was admitted to our set-up on June 29, 2019, for surgery in a case of carcinoma of the larynx post chemoradiotherapy (CRT) six months back at another set-up. The patient complained of hoarseness of voice for the past nine months and of a painless neck mass for eight months, which was gradually increasing in size without redness or restriction in neck movement. The patient was a known case of hypertension for the past 14 years, which was moderately controlled by amlodipine (5 mg BD). He had an episode of ischemic stroke five years ago, and he had been taking clopidogrel (75 mg OD), aspirin (81 mg OD), and atorvastatin (80 mg OD) since then. The patient admitted to having no history of smoking or alcohol intake. The patient’s family history was insignificant.

On admission, the patient’s vitals were stable, and the pre-anesthetic workup ordered by the anesthesiologist revealed low hemoglobin (10.3 g/dL), low red blood cell count (4.1 × 106/mm^3^), low packed cell volume (30%), high creatinine (1.36 mg/dL), and high potassium (5.2 mEq/L; reference range: 3.5-5.1 mEq/L). Other investigations were within the normal range. An electrocardiogram (ECG) and echocardiography showed sinus bradycardia with no other abnormalities. The general physical examination revealed pallor, an enlarged right supraclavicular lymph node, and a non-palpable right carotid artery. Other peripheral pulses were palpable. The patient's American Society of Anesthesiologists Physical Status (ASA-PS) was grade III.

The patient had undergone bilateral carotid doppler study and multi-detector computed tomography (MDCT) carotid angiogram five years back when he had suffered a stroke. The studies revealed that the right common carotid artery (CCA) was totally occluded at the bulb and there was no flow in the right internal carotid artery (ICA). The left CCA showed a 57% diameter reduction. Repeat bilateral carotid doppler study in April 2019 (Table 1) showed a complete thrombus in right CCA from origin to the bifurcation causing 80% luminal compromise and two calcified plaques in the left CCA causing 55% luminal compromise. There was increased anterograde flow in both vertebral arteries due to distal narrowing.

**Table 1:** Chronology of the patient's illness and his management LMWH: low molecular weight heparin, MDCT: multi-detector computed tomography, PET-CT: positron emission tomography and computed tomography.

Date	Important events
2005	Diagnosed with hypertension
2014	Suffered from a stroke, was diagnosed with common carotid artery block
October 2018	Developed complaint of hoarseness in voice
November 2018	Noticed a painless neck mass
January 2019	Underwent chemoradiotherapy for Ca Larynx
April 12, 2019	Repeat MDCT carotid angiography, bilateral carotid doppler study
April 19, 2019	DLscopy (direct laryngoscopy) biopsy
June 20, 2019	PET-CT
June 24, 2019	Stopped clopidogrel and started LMWH
June 29, 2019	Admitted at our set-up for surgery
July 1, 2019	Underwent surgery for carcinoma of the larynx
July 4, 2019	Patient discharged
September 4, 2019	Follow-up

**Figure 1:**
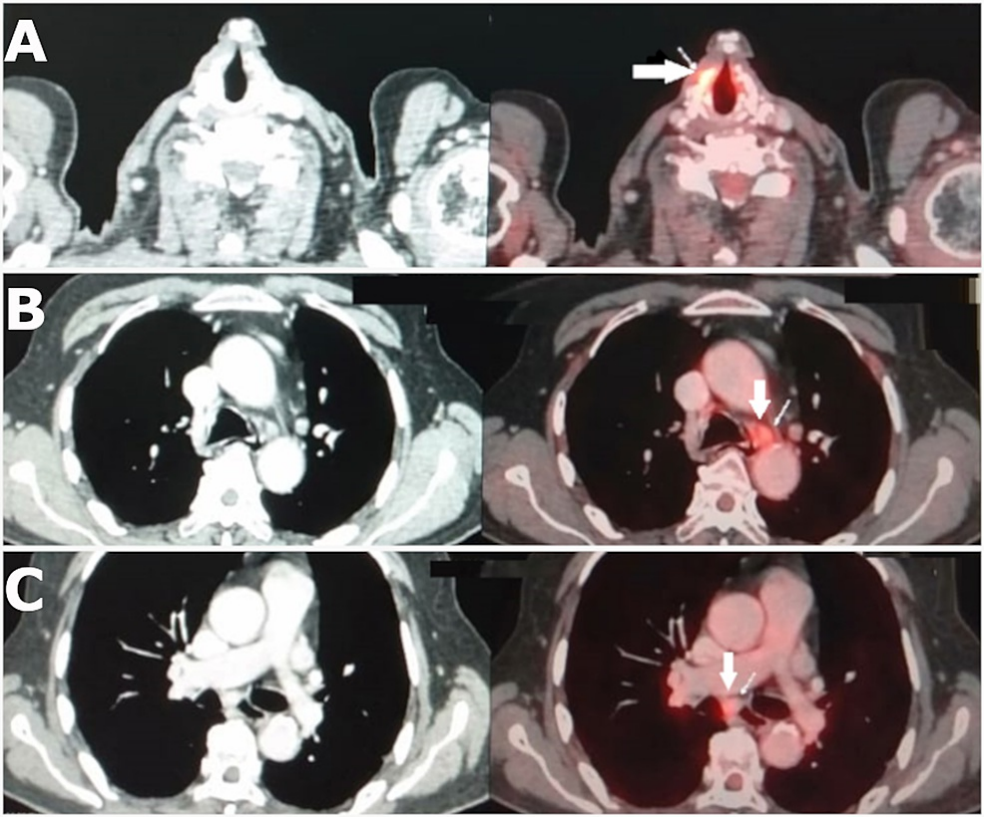
PET-CT for the spread of the carcinoma of the larynx (A) Transaxial CT and fused PET-CT images showing focal FDG avidity along anterior half of right vocal cord (arrow); (B) transaxial CT and fused PET-CT images showing mild degree FDG avid left lower paratracheal node (arrow), which appeared nonspecific reactive in nature; (C) transaxial CT and fused PET-CT images showing mild degree FDG avid subcarinal node (arrow), which appeared nonspecific reactive in nature. FDG: fluorodeoxyglucose, PET-CT: positron emission tomography and computed tomography.

After the routine investigations, a direct laryngoscopic biopsy was performed and the histopathological report confirmed the presence of well-differentiated squamous cell carcinoma of the keratinizing type. Positron emission tomography and computed tomography (PET-CT) revealed increased fluorodeoxyglucose (FDG) uptake in the anterior half of the right vocal cord with a maximum standardized uptake value (SUVmax) of 6.3. A diffuse increase in FDG uptake was seen in the left vocal cord with a SUVmax of 4.1, which appeared physiological in nature. FDG uptake was also seen in the metabolically active right supraclavicular node measuring 0.5 × 0.8 cm^2^ with a SUVmax of 3.7 and the prominent right upper paratracheal node measuring 0.1 × 0.6 cm^2^ with a SUVmax of 7.2 (Figure 1). Hence, total laryngectomy with partial pharyngectomy, bilateral modified neck dissection, central compartment clearance, and permanent tracheostomy was scheduled for July 1, 2019 (Table 1). According to the intraoperative frozen section histopathological report, out of the 13 lymph nodes examined, none showed neoplastic involvement.

We stopped clopidogrel seven days before surgery and started low molecular weight heparin (LMWH) on a prophylactic dose as advised by the physician and neurologist (4000 units of injection Dalteparin given subcutaneously once a day). On the day before surgery, the patient was kept nil-by-mouth overnight for eight hours with an ongoing maintenance intravenous (IV) infusion of 0.45% saline at 2 mL/kg/hour. On the day of the surgery, after shifting the patient to the operating theatre in the morning, we secured an 18-gauge IV line and attached a monitor to the patient to record his blood pressure (non-invasive), SpO_2_, respiratory rate, heart rate, end-tidal CO_2_ (etCO_2_), agent gas monitor (AGM), ECG, temperature (oropharyngeal), and peripheral nerve stimulator (PNS). Foley's catheter was inserted to monitor urine output.

Induction of anesthesia

The patient was pre-oxygenated for five minutes with 100% oxygen. Premedication was done with fentanyl (2 mg/kg/hr), midazolam (1 mg), glycopyrrolate (0.2 mg), and ondansetron (8 mg). The patient was induced at 9:00 am with thiopental (200 mg IV slowly) followed by succinylcholine (100 mg). Nasal intubation with a north nasal Ring-Adair-Elwyn (RAE) tube (no. seven) under the guidance of video laryngoscopy (no. 3 non-channel blades, Ambu India Pvt. Ltd., New Delhi, India) was uneventful.

Maintenance of anesthesia

Anesthesia was maintained with a 40:60 mixture of oxygen and air along with sevoflurane, and relaxation was maintained with atracurium (a 50 mg loading dose followed by a maintenance dose of 0.2 mg/kg/hr throughout surgery by continuous infusion). Intra-operatively, the patient was hemodynamically stable. Hypotension was prevented by keeping the mean arterial pressure (MAP) at more than 90 mmHg. Fluid (0.45% saline) was given at the rate of 6 mL/kg/hr, and urine output was well-maintained at 50 mL/hr. Total blood loss during the surgery was 210 ml, and crystalloids given to the patient amounted to two liters. We kept the dobutamine and norepinephrine drips ready in case hypotension occurred; however, its need did not arise. Ventilation was maintained with a tidal volume of 300 ml, respiratory rate of 14 breaths/minute, inspiration/expiration ratio of 1:2, (fiO_2_) = 40 with closed-circuit ventilation and low flow anesthesia. During the operation, at the time of permanent tracheostomy, the nasal RAE tube was changed to the tracheostomy tube.

Reversal of anesthesia

The reversal was achieved at 2:00 pm with neostigmine (0.05 mg/kg) and glycopyrrolate (0.004 mg/kg) administered very slowly, and the complete reversal was confirmed with peripheral nerve stimulation (PNS). The patient was fully awake, conscious, cooperative, well oriented to time, place, and person without any CNS abnormalities or signs of any neurological damage, and was shifted to the intensive care unit (ICU).

The patient was discharged on the fourth postoperative day (POD) without any complications. Injection Dalteparin (4000 IU OD subcutaneous), atorvastatin (80 mg), and thrice-daily nebulization were started from the first POD onwards. On the second POD, the patient was started on a liquid diet, and physiotherapy was started as the patient complained of difficulty in the neck and upper arm movement. On the third POD, the patient was shifted to the ward from the ICU. Post-operative neurology consultation was not needed as there were no neurological deficits. The patient was called for follow-up one week later to check for surgical wound discharge, and a review of the medication was done by the consulting physician on the date of restarting Aspirin and Clopidogrel. On follow-up after two months, the patient was assessed for alleviation of his presenting complaints (Table 1).

## Discussion

Any surgery requiring the administration of general anesthesia in a patient who has a known case of carotid stenosis falls under high risk and must be performed under caution. Risk factors in our patient comprised carotid artery stenosis, the patient being on clopidogrel, a history of stroke, high creatinine and potassium levels, a medical history of hypertension, and a history of radiation to the head and neck region. Before planning the surgery, we advised the patient to go for carotid endarterectomy, which is a moderate-risk procedure with a MACE (major adverse cardiac events) risk of 1-5%. However, the patient did not consent to the procedure. To maintain cerebral perfusion during surgery, hypotension was avoided in the patient by using thiopental sodium (200 mg IV slowly) instead of propofol for induction, and a 0.45% saline drip was maintained throughout the surgery to keep the MAP at more than 90 mmHg. We stopped clopidogrel seven days before surgery and started LMWH on a prophylactic dose as advised by the physician and neurologist (4000 units given subcutaneously once a day). Due to the patient's high creatinine levels, we avoided using nephrotoxic drugs. As a consequence of high potassium levels, we refrained from using IV fluids containing potassium; hence, we used 0.45% saline rather than ringer lactate (Hartmann's solution), which we normally use at our set-up. Since we anticipated difficult intubation as a result of past radiation to the neck, we used succinylcholine during intubation as its benefits outweighed the risk of further increasing serum potassium in this situation [13]. Anti-hypertensives were given on the day of the surgery, and the patient was kept under strict hemodynamic control throughout the duration of the surgery. Due to a history of radiotherapy and multifocal lytic-sclerotic lesions found on MDCT of the neck, we anticipated difficult intubation and, therefore, kept the fiber optic cable ready. With these precautions, we managed to do a successful operation on this patient and also received a good outcome.

## Conclusions

General anesthesia in a patient with carotid artery stenosis allows us to control the ventilation and airway of the patient, but anesthetic agents reduce cardiac output and blood pressure. Thiopental has neuroprotective effects, while ketamine and nitrous oxide cause short- and long-term damage to brain cells by increasing brain metabolism and oxygen demand. Taking appropriate precautions in high-risk patients in terms of replacing clopidogrel with LMWH before surgery, using thiopental sodium instead of propofol for induction, maintaining an intraoperative MAP higher than 90 mmHg, avoiding nephrotoxic drugs, and using 0.45% saline instead of ringer lactate is crucial for a successful operation and good prognosis, as in our case.

## References

[1] Escalona Belmonte JJ, Biteri Martínez De Iturrate A, Guerrero Orriach JL (2016). Carotid stenosis and anaesthesia. International Journal of Surgery and Surgical Procedures.

[2] Liapis CD, Kakisis JD, Kostakis AG (2001). Carotid stenosis: factors affecting symptomatology. Stroke.

[3] Inzitari D, Eliasziw M, Gates P, Sharpe BL, Chan RK, Meldrum HE, Barnett HJ (2000). The causes and risk of stroke in patients with asymptomatic internal-carotid-artery stenosis. North American Symptomatic Carotid Endarterectomy Trial Collaborators. N Engl J Med.

[4] Raju I, Fraser K (2011). Anaesthesia for carotid surgery. Anaesth Intensive Care Med.

[5] Unic-Stojanovic D, Babic S, Neskovic V (2013). General versus regional anesthesia for carotid endarterectomy. J Cardiothorac Vasc Anesth.

[6] Thompson JE, Talkington CM (1976). Carotid endarterectomy. Ann Surg.

[7] Waltz AG, Sundt TM Jr, Michenfelder JD (1972). Cerebral blood flow during carotid endarterectomy. Circulation.

[8] Sundt TM Jr, Sharbrough FW, Anderson RE, Michenfelder JD (1974). Cerebral blood flow measurements and electroencephalograms during carotid endarterectomy. J Neurosurg.

[9] Hino H, Matsuura T, Kihara Y, Tsujikawa S, Mori T, Nishikawa K (2019). Comparison between hemodynamic effects of propofol and thiopental during general anesthesia induction with remifentanil infusion: a double-blind, age-stratified, randomized study. J Anesth.

[10] Harris CE, Murray AM, Anderson JM, Grounds RM, Morgan M (1988). Effects of thiopentone, etomidate and propofol on the haemodynamic response to tracheal intubation. Anaesthesia.

[11] Apinis A, Sehgal S, Leff J (2014). Intraoperative management of carotid endarterectomy. Anesthesiol Clin.

[12] Kaisti KK, Långsjö JW, Aalto S (2003). Effects of sevoflurane, propofol, and adjunct nitrous oxide on regional cerebral blood flow, oxygen consumption, and blood volume in humans. Anesthesiology.

[13] Schow AJ, Lubarsky DA, Olson RP, Gan TJ (2002). Can succinylcholine be used safely in hyperkalemic patients?. Anesth Analg.

